# A Review of Multisensor Data Fusion Solutions in Smart Manufacturing: Systems and Trends

**DOI:** 10.3390/s22051734

**Published:** 2022-02-23

**Authors:** Athina Tsanousa, Evangelos Bektsis, Constantine Kyriakopoulos, Ana Gómez González, Urko Leturiondo, Ilias Gialampoukidis, Anastasios Karakostas, Stefanos Vrochidis, Ioannis Kompatsiaris

**Affiliations:** 1 Information Technologies Institute, Centre for Research and Technology Hellas, 6th km Charilaou-Thermi Road, 57001 Thessaloniki, Greece; evanbekt@iti.gr (E.B.); kyriak@iti.gr (C.K.); heliasgj@iti.gr (I.G.); akarakos@iti.gr (A.K.); stefanos@iti.gr (S.V.); ikom@iti.gr (I.K.); 2 Ikerlan Technology Research Centre, Basque Research and Technology Alliance (BRTA), P^o^. J. M^a^. Arizmendiarrieta 2, 20500 Arrasate-Mondragón, Spain; ana.gomez@ikerlan.es (A.G.G.); uleturiondo@ikerlan.es (U.L.)

**Keywords:** smart manufacturing, data fusion, feature extraction, industrial prognosis

## Abstract

Manufacturing companies increasingly become “smarter” as a result of the Industry 4.0 revolution. Multiple sensors are used for industrial monitoring of machines and workers in order to detect events and consequently improve the manufacturing processes, lower the respective costs, and increase safety. Multisensor systems produce big amounts of heterogeneous data. Data fusion techniques address the issue of multimodality by combining data from different sources and improving the results of monitoring systems. The current paper presents a detailed review of state-of-the-art data fusion solutions, on data storage and indexing from various types of sensors, feature engineering, and multimodal data integration. The review aims to serve as a guide for the early stages of an analytic pipeline of manufacturing prognosis. The reviewed literature showed that in fusion and in preprocessing, the methods chosen to be applied in this sector are beyond the state-of-the-art. Existing weaknesses and gaps that lead to future research goals were also identified.

## 1. Introduction

The advances in Information Communication Technologies (ICTs), along with the era of Industry 4.0 have brought industries closer to the adoption of automation in their processes. Industry 4.0 is formed [1] by the integration of manufacturing operation systems and ICTs. Business models are reframed by adopting the digitisation and Internet of Things (IoT) concepts. The IoT refers to the devices and sensors that are smart, are addressed using communication protocols, are autonomous, and also adapt to the operating conditions [2]. The availability and variability of sensors assist in this transition to automation, by monitoring the function of machinery and tools, as well as measuring the product quality. This intends to improve the failure ratio of machines and products, maximizing the productivity of a company and a good machine function. Along with the increasing usage of sensors in manufacturing procedures comes the need to exploit the large amounts of heterogeneous data they produce and, subsequently, the application of fusion techniques that combine these data with different methods and improve the knowledge extracted from them.

Fusion can be described as the method of combining data from different sources. In the field of machine learning, there are two broad categories of fusion: early and late fusion. Early fusion is implemented in the first stages of an application and refers to the combination of the raw data (data fusion) or the extracted features (feature fusion). Late fusion refers to the combination of the results of different algorithms and is implemented as the last step of the analysis’ pipeline. Late fusion can be accomplished with techniques that combine the predicted class probabilities of each algorithm or the predicted classes of each algorithm. More advanced techniques such as stacking and boosting use the output of other classifiers as the input to a new ensemble algorithm.

This paper provides a review of data fusion solutions implemented in the manufacturing sector, with a special emphasis on the sensors employed in the industry, as well as the preprocessing stages of data preparation and feature extraction. Furthermore, the types of data storage and techniques for data extraction used for industrial prognosis are explored. The aim of this literature review is to provide the reader with an introductory guide for the processing of industrial data, with a special focus on the types of fusion employed in industrial prognosis in recent years.

The methodology followed in order to collect the papers included searching in some popular databases such as Scopus, ISO, and Google Scholar. Using keywords, which are mentioned below, combined with the term “manufacturing” or more specific terms relevant to manufacturing applications, e.g., chatter detection, some papers were collected and screened for relatedness to the scope of the current literature review. Papers published in recent years, describing the architecture or the analytical framework of manufacturing problems, were selected. The majority of papers reviewed were published after 2015, with some exceptions that were published in early 2000. Following is the list of keywords used for some of the main sections of the paper:

- “Vibration analysis, current signature, acoustic analysis, thermography, infrared radiation, thermocouple, acoustic emission, NDT, defect inspection, ultrasound, induced current, radiography, penetrating liquids, lubricant analysis, ferrography, spectrometric, chromatography, vibration processing, motor current signature analysis, feature extraction, feature selection, dimensionality reduction, PCA, mRMR, acoustic camera, 3d camera, artificial vision” combined with the term “manufacturing”, for the section of data preprocessing;
- Data fusion solutions/applications in manufacturing, ensemble learning in manufacturing applications, fusion for chatter detection, fusion for tool wear detection, and data fusion in industrial prognosis, were some of the keywords used for the section of data fusion solutions in manufacturing.

**Figure 1:**
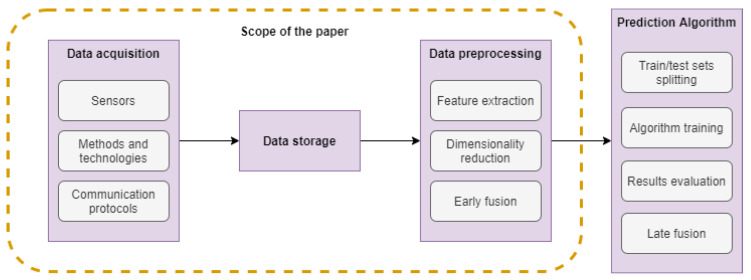
Flowchart that presents the typical analytic pipeline followed in manufacturing applications.

The analysis of data collected from different sensors of a typical manufacturing process comprises certain steps, similar to the steps of any analytic task that involves prediction (Figure 1). In the beginning, raw data need to be collected from sensors and stored. In order for the data to be analysed, some cleaning and filtering functions are usually applied. Following is the important step of filtering, which removes signal noise, and afterwards, features are usually extracted. Feature engineering is crucial in signal processing, and the transformation methods adopted, as well as the features extracted vary according to the type of sensor and the goal of the analysis. The large amount of features produced is then reduced by applying feature selection methods, which select smaller sets of variables for input in a classification or regression algorithm. Fusion can be applied in different stages of the aforementioned pipeline, whether in the beginning combining the raw data, or after the step of feature extraction to combine the features, or even in the final stage for the combination of the prediction algorithm’s results. In the current work, more attention was given to the initial layers of the analytic pipeline, reviewing the types of preprocessing used in industrial prognosis problems and the common useful features produced. Furthermore, the fusion techniques adopted in manufacturing prognosis were reviewed. Thus, the scope of the paper is depicted in Figure 1), responding to the sections included in the dotted lines, while the figure overall describes an analytic pipeline.

Similar literature reviews regarding fusion in the era of Industry 4.0 have been published in recent years. A multisensor data fusion review was presented in [3], with a special focus on multisensor monitoring technology and the architecture of multisensor monitoring systems. An analytical survey on machine learning and fusion applications for industrial prognosis was presented in [4]. The authors provided a categorisation of methods according to the cause of the failure to be predicted. This review referred to the general industry section, while our current literature review paper presents the fusion trends in manufacturing applications. In [5], a review of the techniques used for data fusion for material data was presented. The paper reviewed the methods and underlying theory for material data fusion, explaining the different levels of fusion and how these can be adapted in the combination of heterogeneous material data. The authors also gave insight into the differences of the terms “data integration” and “data fusion”, which are often met in manufacturing applications.

The rest of the paper is organised as follows: Section 2 describes the methods for data storage and extraction, along with the communication protocols enabling the fusion systems. Section 3 refers to the type of sensors employed in manufacturing, data preprocessing techniques, and feature extraction, as well as feature selection methods. Section 4 reviews the fusion methods adopted in different systems and in the manufacturing sector specifically. Finally, the paper is concluded with the Discussion Section.

## 2. Data Acquisition and Storage

The evolution of technology and especially the Internet of Things (IoT) has led to a new kind of manufacturing known as Smart Manufacturing. Smart Manufacturing is an application of the IoT that focuses on using inexpensive, small-sized, and smart devices that are all interconnected so that they can increase productivity and improve the health of the machines. Big Data in Smart Manufacturing systems are continuously generated data in high volumes produced by said smart devices [6] and are available in various forms, e.g., log files, signal streams, or sensor data. A Big Data analysis system should be able to use these data in real time, as well as save them for historical analysis and long-term pattern detection. This section of the paper deals with the ways data are acquired and stored from such smart devices.

### 2.1. Data Acquisition Methods and Technologies

In [7], a comprehensive review of Big Data analytics throughout the product lifecycle was made. Most notably, regarding the data acquisition phase of the lifecycle, it was acknowledged that manual-based data acquisition methods are still used in various stages of the lifecycle process, thus making the acquired data from these approaches inaccurate and untimely, and as a consequence, the decisions based on them are usually ineffective. The authors [8] suggested some challenges of data acquisition that need further research to be resolved and used smart mobile devices to provide an example on how IoT technologies can be embedded into the physical world and be able to gather data throughout the whole product lifecycle. A detailed hierarchical architecture of a smart factory was described by [9], emphasizing the need for Wireless Sensor Networks (WSNs) in a smart factory for data monitoring, acquisition, and logging. ZigBee and Bluetooth were also mentioned, on top of Radio-Frequency Identification (RFID), for real-time data acquisition and were described as good choices when it comes down to the cost of the industrial automation of wireless technologies. Furthermore, the devices responsible for data acquisition should be easy to set up and connect with interfaces capable of scaling up.

One methodology employed by [10] was a monitoring tool organised in a WSN. Part of the monitoring tool is a Data Acquisition Device (DAQ) that uses split-core current transformers, closed-loop Hall effect sensors, and a camera to create an easy and not intrusive way to collect data by monitoring the status of the machines. The researchers of the paper used multiple DAQs on the shop floor and used a central gateway to collect and organise the data into packets before they were transmitted. The WSN that was used for data extraction utilised the DIGI XBee ZigBee Radio-Frequency (RF) module. As the paper described, an OPC Unified Architecture (OPC-UA) was used. The OPC-UA provided an extensible data model, which provided the data schema. A NoSQL database was used, as it proved to be more flexible than a Structured Query Language database (SQL) because of the heterogeneity and the different data that were being generated. The authors of [11] create a Cyber–Physical System (CPS) that uses a semantic sensor network. Focus was given on the way data are gathered from the physical sensors. To manage the large volume and velocity of the data, the authors proposed an architecture in which the data are collected through an OPC-UA, an industrial M2M communication protocol. There exists a considerable body of literature on data flow models, but most notably, Reference [12] suggested frameworks that allow users to model their application via visual editors. These programs enable the users to receive data from external sources such as IoT devices and smart sensors.

In [13], the authors suggested an architecture design for a smart manufacturing system. Furthermore, they provided detailed considerations of the way a smart Manufacturing Execution System (MES) should be designed. For real-time data acquisition from the shop floor, the OPC-UA technology was proposed. The authors of [14] expanded on the topic of data transmission with the introduction of WiFi direct, 4G LTE, and Z-Wave. There was also the mention of the protocols being used with these wireless technologies, which include IPv6, MQTT, SOAP, and REST, among others. The authors also mentioned a series of compatible with Supervisory Control And Data Acquisition (SCADA) communication networks such as OPC, Open Database Connectivity (ODBC), RS232, and Dynamic Data Exchange (DDE), as well as some wireless communication standards such as the Highway Addressable Remote Transducer (HART) and ISA100.11a. The most prominent of these protocols are the MQTT and REST API. The MQTT protocol is used to acquire and transmit data from large industrial environments to the cloud where the processes producing the data can be monitored and controlled. The REST Application Programming Interface (API) provides a way to securely collect data from the IoT devices, where the data are collected in formal message arrays and the receivers split those individual messages so the producing device can be identified. SCADA systems provide a fully connected system that a manufacturer can use not only to acquire the data, but also to handle, manage, and archive them long term. Some known SCADA systems are SIMATIC SCADA Systems from Siemens, AVEVA™ Plant from Schneider Electric, Proficy HMI/SCADA from General Electric, and HMI/SCADA from ABB.

A demonstration of real-time data acquisition using the MQTT protocol was described in [15]. The authors implemented a system using temperature and humidity sensors so they could generate data to work with the protocol. For the test, they generated data for 60 s and compared the ability between the Hypertext Transfer Protocol (HTTP) and the MQTT protocol to transfer from the hardware (i.e., sensors) to the server and store them in a MySQL database. To minimise the error and loss of data, each transmission had a sequential ID so the completeness of data could be checked. A conclusion was made that the use of the MQTT protocol proved to be up to six-times faster than HTTP at sending data. On the more technical side, it was reported in [16] that depending on the application and the network coverage required to send data different, protocols may need to be used. Low-Energy (LE) Bluetooth, Near-Field Communications (NFC), and RFID, among others, are technologies used for short-range communication. As a result, industrial applications that require a broader field to be deployed need solutions that can be both energy saving while maintaining a significant coverage area. Such a technology is the Low-Power Wide-Area Network (LPWAN), which includes Sigfox, LoRa, and the Narrowband IoT (NB-IoT) [17].

A Big Data pipeline for data streaming in Industry 4.0 was described in [14,18]. Moreover, data collection and data storing tools were compared and presented. Such tools are Apache Kafka, RabbitMQ, and Amazon Kinesis, which are considered for pushing a high volume of messages that are produced from data producers (i.e., sensors) and even Apache Storm to process and discard “useless data”, which were tagged as less important or out of context.

In [19], a system that is capable of data monitoring and acquisition of a Computerised Numerical Control (CNC) machine tool in intelligent manufacturing was proposed and developed. Most notably, the authors compared the different data acquisition methods from a CNC machine, not only on the different data types that can be collected, but also the technical difficulty and implementation costs. For data acquisition, the MTConnect protocol was selected, while for the database, a system that uses the ODBC method was the choice. The machine tool networking was based on an industrial Ethernet and Transmission Control Protocol/Internet Protocol (TCP/IP) technology. Working also with CNC machines, Reference [20] provided a thorough explanation of the way a CNC machine generates data and how these data are acquired, transmitted, and stored. CNC data can be split into two main sources: controller data and external sensor data. As is usual for the sensors, the collected data contain noise from external interference, and for that reason, a necessary step is to clean and preprocess the data. For machines such as a CNC system, a high amount real-time data in the controller is required. Some of the most-used technologies in the field are Ethernet, Profinet, and EtherCAT, but in order for them to work seamlessly in the system, the sensors need to be equipped with acquisition cards.

Previous research showed that data acquisition in real time is based on the configuration of the smart environment [21,22,23]. Specifically, it was described that the first part of the data acquisition is the data collection, during which raw data are gathered using various technologies depending on the application. Moreover was proposed that Ultra-High-Frequency (UHF) RFID readers can be used to track and collect data in real time from the manufacturing process. Regarding the transmission of the collected data, it was mentioned that for real-time data such as temperature, vibration, and pressure, the Internet, wireless, and 4G methods were used. As far as non-real-time data (e.g., maintenance history) transmission are concerned, tools such Sqoop are preferred. A more in-depth look at the way RFID technology is used to collect data from the shop floor was provided by [24,25], where the authors explained step by step the system architecture they created. Data flow starts from RFID tags, which are attached to the input and output of their manufacturing section. This allows for real-time monitoring of the manufacturing process and can update individual data from each part. The authors concluded that such an architecture (RFID-based IoT solution) with the ability to closely monitor the manufacturing sections leads to improvements in the traceability, quality, and tool wear prediction.

Some authors [26,27] have also suggested Industrial Internet of Things (IIoT) architectures, where the data collection method was thoroughly described. Specifically, all kinds of manufacturing data (e.g., equipment status data, product data, or measurements) can be gathered using wired or wireless methods. The wireless methods include, mostly, as previously mentioned, RFID readers that obtain the raw data. Hive and HBase have been introduced as a distributed data storage system. One way that is considered for data transmission is the Flume interface, which forwards the data to a selected storage system.

### 2.2. Data Storage Software Solutions

Concerning data storage, a distributed approach is usually the choice where a Distributed Database System (DDBS) is used to store structured data and the Hadoop Distributed File System (HDFS) or NoSQL databases for unstructured data. Other alternatives are the C Open Source Managed Operating System (COSMOS) and Haystack. Especially for distributed file systems, the Google File System (GFS) was one of the first systems developed to handle data-intensive applications.

MongoDB, a NoSQL database, is one of the most popular databases at the moment, and the authors of [10] used it to store the sensor data that were gathered by the DAQs. As was mentioned by [13], relational databases such as MySQL are not an option due to the complicated nature of the manufacturing data. Subsequently, the authors considered a distributed database as an appropriate option because of its high performance, efficiency, and scalability.

Regarding data storage solutions being proposed, the authors in [18] listed, described, and compared commonly used technologies such as Hadoop Hive and MongoDB. A distinction was made between the data models that were used: firstly, the file system data model, for data stored in a schema-less manner and read in a structured manner with a processing time based on the processing needs of the application; secondly, document-based data model; lastly, a column-based schema. A reference was made regarding the recent data storage technologies and their capabilities to process data for critical real-time applications.

Prior research [28] suggested that shop floor data on a manufacturing site can be gathered using what is called a SCADA system. SCADA provides a singular interface, where all the gathered data collected from different smart devices can be transmitted. Among SCADA, Reference [29] mentioned the Protocol Data Unit (PDU) as an alternative data acquisition tool. It was also suggested that the combination of IoT and cloud services gives the ability for different equipment to be connected and collect huge amounts of data. To organise data in a methodical and effectual way, Database Management Systems (DBMSs) have been created. According to [22], these tools can be split into two categories, relational DBMS and non-relational DBMS. The first category includes SQL databases, meaning databases that usually store data in tables of records. Commercial solutions for SQL databases are Microsoft SQL, PostgreSQL, Oracle, and MySQL. Regarding NoSQL databases, it is possible to use various types of data such as text, binary, and records. One of the benefits of NoSQL over SQL is that it is scalable and can support huge volumes of data, making it perfect for managing data coming from sensors and smart devices. In [30,31], a straightforward software solution explaining the pros and cons of each one was provided. The most commonly used solution is Apache Hadoop, but the authors [30] also proposed other options such as: Redis, SimpleDB, CouchDB, and MongoDB, just to name a few. More research [32] described the criteria of the data model and used them to identify different models where each of the previously mentioned software solutions apply the best.

Authors such as [33] provided a more technical side to the way data being acquired and stored. Even though it was mentioned that the data were collected manually, there was an in-depth review of the storage methods. As has been previously reported by the literature [34], the most adequate NoSQL databases for real-time data storage are HBase and Cassandra. However, it was demonstrated by [35] that Online Transaction Processing (OLTP)-oriented NoSQL databases can lack the support for fast sequential access over a significant amount of data, which sometimes can prove to be a hurdle when it comes to data analytics. Hadoop BDW is proposed as the opposing solution that can handle fast sequential access.

A data storage framework was presented in [36] that can deal with various types of data collected from different devices, for instance RFID readers, monitors, or thermometers. Due to the heterogeneity and volume of data, there is no perfect method for efficiently storing and accessing them. The proposed architecture by the authors included several modules. In more detail, HDFS was used for unstructured file storage, while a database module using NoSQL and relational databases was used to manage the structured data. The authors also investigated a data storage framework capable of being a feasible solution to challenges such as a large volume of data, different data types, rapid generation of data, and the complicated requirements of data management. In detail, for structured data, a database management model was created that combined and extended multiple databases. For unstructured data, a common solution was followed. The authors explained in great detail how the framework extended HDFS for multitenant data isolation. Concluding, it was mentioned that for remote and cross-platform data access, a RESTful-service-generating mechanism was integrated, to provide a platform-independent HTTP interface. Furthermore, the authors of [37] reported that in large-scale manufacturing systems, tens of thousands of data streams flow into the storage at various rates. For that reason, there is a need for improving the bottlenecks that cause the data to arrive irregularly. The authors mentioned Blueflood as a solution that attempts to achieve high scalability. Blueflood is a combination of Cassandra, which handles data storage, and Elasticsearch, which handles indexing. A similar alternative to Blueflood is OpenTSDB, which uses HBase for storage instead of Cassandra. A summary of the software tools used for data management can be found in Table 1.

The authors of [38] separated the requirement and solution components that are assigned to the data storage processes. In [39], it was suggested that due to comprehensive process transparency, structured, semi-structured, and unstructured data should be stored and made available for application-specific processing. Furthermore, in [38], operational data storage and a long data storage system were proposed. The first one requires an edge device unit, which must be able to store and manage real-time data. For operational data storage, the edge devices need to store data efficiently and reliably. For that reason, SQL was recommended. The preferred Relational Database Management Systems (RDBMSs) are MySQL and PostgreSQL. Concerning long-term data storage, a Big Database system is required. Consequently, NoSQL databases provide the best storage solutions as they are able to efficiently store large volumes of unstructured datasets, compared to relational databases [30,40].

A focus on data storage issues and recommendations for a new solution to organise and manage data was given by [41]. In particular, the cloud storage system they presented uses a Document-Oriented Storage System (DO-SS) for the storage of all the information derived from the monitoring systems. The integration between the data collection and storage subsystem occurs with the help of a software module (parser), so that the data can be converted before being stored in MongoDB. The authors also implemented an Object-Oriented Storage System (OO-SS), a widely used object storage system. The main benefit over other solutions, is that the data are protected by being stored as multiple copies, so in case a node fails, there is another one active where the data are stored. This design makes the OO-SS ideal when there is a need for performance and scalability.

In [42], a hybrid framework was conceptualised for an industrial platform that ensures efficient and accurate communication, concerning data transfer among software applications and devices. The framework was characterised as hybrid, because it contains two different technologies for data storage and exploits the best features from each of them. In the proposed framework, structured data are stored in relational database systems, while sensorial data, which most of the time tend to be unstructured, are stored in NoSQL databases. The real-time sensor data are published to an MQTT broker that is suitable to be used to connect with remote locations, and the Raw Data Handler subscribes to MQTT and acquires the generated data. Later, the data are carried to the sensorial repository where they are indexed and can easily be filtered and accessed by timestamps. In general, the authors proposed a hybrid framework that is capable of shop floor data collection and application in industrial environments.

A scalable data storage framework for smart manufacturing was introduced by [43]. In more detail, it is a Software-Defined Hybrid Cloud (SDHC) for saving the equipment-generated data. The main challenge the authors faced was the different data types and formats. With the use of software-defined technology, control data and manufacturing data were separated. The manufacturing data derived from the sensors and controllers can be saved in a key-value database only if the data save request is allowed. Lastly, two improvements were proposed, the first one to deal with the data saving efficiency, which will improve the response time, and the second one with the data-save permission, which will improve the system’s robustness. A software architecture was designed by [44]. The framework is highly scalable, so a fleet of IoT boards and sensors can be easily configurable. For data collection, an Arancino board was used that was provided with an AI module that can manage on-board fault prediction. InfluxDB was the database that was selected as it is non-relational and is suitable for industrial scenarios where sensors send data at different rates.

**Table 1:** Applications of software tools for data management.

Software Tool	Application	Reference
Apache Hadoop	Hadoop is a framework that allows for the distributed processing of large datasets across clusters of computers using simple programming models. It has been used for different kinds of applications such as frameworks that can optimise and organise the way bit data can be searched and accessed. There are also applications regarding storing data derived from sensors that monitor the environmental air pollution. Lastly, tuning systems have been designed to improve the performance of Hadoop and MapReduce.	[45,46,47]
Apache Storm	One of the most capable software solutions for Big Data is Apache Storm. Several applications exist that employ it. Some of them use it as a data streaming and real-time processing platform, while others create frameworks for dynamically scaling for the analysis of streaming data. Finally, there are multisensor data fusion frameworks that employ Apache Storm due to its high reliability and good processing mode.	[48,49,50]
Apache Flume	Flume is a distributed, reliable, and available system for efficiently collecting, aggregating, and moving large amounts of event data. It has been used for various kinds of applications such as healthcare and manufacturing. Frameworks have been designed so that the computational scalability of sensor network data can be achieved.	[51,52,53]
Apache Spark	The aim of Spark is to make data analytics programs run faster by offering a general execution model that optimises arbitrary operator graphs and supports in-memory computing. Most applications use it for sensor analytics. It has been deployed on both industrial and non-industrial applications and can be integrated into pre-existing frameworks.	[54,55,56]
Apache Kafka	Kafka is well suited for the situations where users need to process real-time data and analyse them. There are papers that focused on learning how to reliably transfer data and studied its application in collaboration with other software solutions.	[57,58,59]

### 2.3. Communication Protocols

The smart manufacturing sector benefits from data fusion systems. Next, the required underlying communication technologies enabling the fusion systems are presented. These are related to the IoT and the corresponding protocols. Specifically, networking concepts that are based on software are incorporated into the lower communication layers. Furthermore, adaptivity offers advanced performance since the nature of modern networking systems is dynamic.

Software-Defined Networking (SDN) is the main conceptual networking model [60] under modern IoT fusion environments. It brings the programmable networking logic into the lower architectural layers. This process allows better control and management of networking data flows in a transparent way from the higher-layer networking applications. It is a centralised architecture that defines a stable ground to be used for building networking applications. An open implementation of the SDN networking concept is the OpenFlow protocol [61], which is widely adopted. A networking foundation is currently maintaining the specification. The whole concept relies on central computing logic, represented by the SDN controller, controlling data flows between core networking components such as switches (i.e., the Southbound API). Fusion techniques over IoT environments are facilitated with the exploitation of the SDN concept.

Achieving high-speed transmissions in IoT environments requires efficient and dynamic channel assignment. Conventional fixed assignment techniques are not adequate for modern environments, in which, due to their dynamic nature, requirements must constantly adapt to the runtime conditions. The IoT over SDN, when combined with deep learning techniques, improves transmission quality. Therefore, a traffic load prediction algorithm that is based on deep learning [62] has been proposed, forecasting network traffic and congestion. Next, a deep-learning-based algorithm that assigns channels has been introduced. Its main role relates to link channel allocation using intelligence in the SDN-IoT network environment.

Since communication between smart devices in the IoT manufacturing sector can be peculiar, event-based data fusion for communication is needed [63]. This is a message exchange system between participating devices that initiates when events occur. Fusion is required since different devices generate heterogeneous notifications, along with data source trust issues that may arise. The contribution of this work consisted of an event-based protocol covering the communication issues of resource-limited sensors and heterogeneous data sources, and it considered the trust degree of the fused data.

There is a vast spectrum of IoT applications that require security and privacy for realistic deployment in the modern era. Trust and data integrity are prerequisites in the IoT ecosystem, otherwise applications will lose high demand and also their potential. In the current case of cellular and sensor networks, special security challenges emerge and correlate with authentication issues, privacy, management, and information storage [64]. Programmable Logic Controllers (PLCs) are an integral part of the industrial control systems [65]. Communication issues between PLCs and the engineering stations or field devices concerning security must be confronted. Modern database systems use communication systems to deploy as cloud-based solutions [66]. Different DBs support various security technologies, though most non-relational solutions overlook modern Big Data applications.

Communication requires a credible authentication model, which guarantees data integrity and secrecy. For that purpose, an IoT node-roaming authentication protocol was introduced [67]. A heterogeneous fusion mechanism comprises the protocol’s functionality. Every roaming device communicates with a server, which provides authentication functionality. This process renders attacking attempts from external malicious nodes difficult.

In a smart manufacturing environment, multiple protocols are required for transmitting data efficiently. SDN forms the basis for a heterogeneous network architecture [68] for forwarding multisource manufacturing data and, at the same time, utilising network resources optimally. The core algorithm of the proposed protocol is based on cross-network fusion and scheduling. It was shown that the efficiency was improved for the fusion processes, especially for intelligent manufacturing equipment.

## 3. Data Preprocessing and Feature Extraction

### 3.1. Types of Sensors and Variables Measured

In the context of Industry 4.0, the use of sensors has been widely extended, as the capacity to store and use the acquired data has been enhanced [69]. The heterogeneity of the machinery in the manufacturing plants and the specific needs of each sector generate the need to use different kinds of measurements to monitor the machines, the manufacturing processes, and the parts that are produced. For that purpose, a large number of sensors are available on the market. Many of them are already integrated in the machines, even if it is usual to include other sensing equipment in order to complete the measurement chain available for further analysis.

In [70], some general guidelines were given for the condition monitoring and diagnostics of machines. The parameters that can be measured were identified and classified according to the type of machine that was considered. The main parameters that are useful for all kinds of machines are: temperature, noise, vibration, acoustic emissions, ultrasonics, oil pressure, and thermography. Some others will be specific for some particular types of machines. In the next paragraphs, this is explained in more detail.

Vibration analysis is a commonly used technique [44,71,72] as the vibration signature of a component may change as a fault develops. Machines vibrate in their normal operation, some of the vibratory phenomena related being to events that occur periodically (such as the rotation of shafts, the mesh of gear teeth, or the generation of rotating electric fields). Thus, the frequency of that vibration often gives an indication of the source. That is why it is important to establish a baseline for the standard vibration response of a machine and detect any anomaly from that baseline when a fault occurs [73]. For that purpose, different kinds of vibration transducers can be used: proximity probes (measuring the relative distance between the probe and another surface), velocity transducers (providing a signal proportional to the absolute velocity of the element on which the transducer is mounted), accelerometers (giving a signal proportional to the absolute acceleration), dual vibration probes (allowing the measurement of absolute motion), and laser vibrometers (transducers based on the Doppler principle that do not load the measurement object) [73], accelerometers being the most commonly used. In the range of the audible spectrum, acoustic analysis via microphones [74] allows the localisation of an internal material transformation or a noise source, but it is hard to reach to determine the magnitude of a failure, given that manufacturing plants can be noisy environments.

Electrical current is usually measured in electrical machines such as motors or generators [44,75]. This kind of signal has a low implementation cost, as the equipment is simple and economical. Other variables to be measured in such rotational equipment are the torque, the speed, and the temperature. As the temperature changes or reaches a limit, the temperature can be a signal of failure, and the analysis of the temperature is another approach [76]. One of the most-used techniques is thermography, which consists of the determination of the surface temperature by means of measuring the infrared radiation [77]. This technique can be used, for example, to monitor the wear of cutting tools [78]. Moreover, thermocouples are commonly installed in machinery thanks to their low cost, providing the value of the temperature in a single point.

The measurement of Acoustic Emission (AE) [79,80] allows capturing the propagating waves generated by the rapid release of energy from localised sources, as a result of crack propagation, impact, or leakage, among other phenomena [81]. In the manufacturing context, this technique can be useful to monitor the state of the structure of large machinery subjected to cyclic loads or to test the quality of a product. Similarly, ultrasound analysis can be used to detect, identify, and evaluate the size of surface and sub-surface failure or to measure material thickness. Thus, it is mainly used in material or product control stages [82]. Induced currents are useful to test electrically conductive materials [83]. They are a means to evaluate surface and sub-surface failure, as well as to measure material thickness generated in processes such as thermal treatments and coatings.

Radiographies are used to detect internal cracks and the lack of homogeneity of materials [84] (e.g., in pieces manufactured by melting or in welded joints); however, access to the analysed object from all possible perspectives is needed, and this is an expensive technique. Penetrating liquids are also used to detect surface discontinuities.

Lubricant analysis is used to determine the chemical composition of lubricants, as well as to find particles in them. Some of these techniques are ferrography, spectrometric identification, and chromatography. Ferrography looks for iron particles in oils in order to identify the component that is wearing out and to determine the wear level [85]. Spectrometric identification looks for iron, metallic, or non-metallic particles by means of the atomic emission spectrometer, which is useful to identify early-stage failure [86]. Chromatography measures changes in the properties of the lubricants (viscosity, pH, water content, etc.) [73].

The aforementioned sensors and variables measured refer mainly to one-dimensional data. However, higher-dimensional data are also relevant in the context of smart manufacturing. Vision techniques can be used to inspect manufactured products [87]. Three-dimensional vision techniques are also useful in inspection environments, such as the analysis of the quality of an assembly process [88]. Vision techniques can be combined with sound measurements using acoustic cameras, imaging devices with an included microphone array. They process sound signals to form a representation of the originating location. They are used for the identification of noise sources in machinery such as conveyor belts [89].

Even if the aforementioned variables are general-purpose, there are many others that can be used in industrial equipment, which depend on the kind of machinery. For example, those equipment related to pneumatic or hydraulic subsystems (valves, cylinders, and so on) may take advantage of the measurement of pressure, flow, position, etc. Moreover, other equipment suffering from cyclic or transient loads may have installed load cells or strain gauges to measure the force or the displacement, which can be useful to calculate the mechanical stress suffered by a component.

### 3.2. Data Preprocessing

Data preprocessing is required in order to treat the data and obtain clean data that can be useful for feature extraction. Even if in some scenarios, features are extracted directly from the raw signals, the high complexity of the industrial systems and the nonlinear processes involved ask for the application of signal processing strategies [90]. These strategies may include the removal of the effects of the operating conditions from the acquired signals, the removal of noise in noisy signals, the identification and removal of outliers that may lead to non-significant features, and the transformation of the signals to other domains from which key features can be extracted, among others. There are many common techniques for some of the variables that can be measured and were introduced in Section 3.1. However, some of them are dependent on the nature of the signal. In this subsection, the focus is put on two of the main data sources: vibration signals and current signatures. This selection was made based on the widely extended use in manufacturing machinery in the case of vibration signals, the ease of data acquisition, the fact of being a usual data source in the manufacturing industry, and its usefulness for the detection of faults and inefficiencies in machines in the case of current signatures.

Vibration data preprocessing is one of the most-studied areas in signal preprocessing. ISO 13373-2:2016 [91] summarises some procedures for general purpose applications. Thus, different effects and techniques are described, such as time domain averaging, Fourier transform, spectrograms, order tracking, octave and fractional octave analysis, and cepstrum analysis. According to the standard, there are other techniques for the in-depth analysis of vibration signals. This is the case of the semi-automated bearing diagnostic procedure [73], which consists of five steps for the processing of vibration signals corresponding to rolling element bearings, with different objectives: (i) removal of speed fluctuation, based on order tracking [92]; (ii) removal of discrete frequencies by means of discrete random separation, self-adaptive noise cancellation, or linear prediction [93,94]; (iii) removal of the smearing effect of the signal transfer path using minimum entropy deconvolution [95]; (iv) determination of the optimum band for filtering and demodulation via spectral kurtosis [96]; (v) transformation of the domain by means of envelope analysis [97]. VDI 3832 [98] also provides some guidelines on the analysis of rolling element bearings in driving and driven machines and power transmission elements. Other advanced techniques comprise empirical mode decomposition [99], Hilbert–Huang transform [100], adaptive local iterative filtering [101], or fast iterative filtering decomposition [102], among others.

Regarding the analysis of current signals, Motor Current Signature Analysis (MCSA) is a common practice to diagnose different rotating machinery, as is the case of induction machines [103], permanent magnet synchronous motors [104], or rolling element bearings [105]. This strategy is also complemented with other signal processing techniques such as adaptive filters such as the Wiener filter [106] or time-synchronous averaging [107].

### 3.3. Feature Extraction

Feature extraction is a key step as this process creates representations of data that increase the effectiveness of the posterior analysis. Thus, the extraction of relevant features for the specific machine or process and the fact of generating features with reduced missing data are important for the accuracy of the models that can be trained afterwards [108]. It should be highlighted that some advanced artificial intelligence models do not need this feature extraction step to be performed previously, as they operate directly with the acquired time series, but these techniques usually require very large data volumes [69]. The feature extraction techniques can be categorised depending on the domain in which the features are extracted [109]. Thus, the features that can be extracted in different domains are presented next.

Time domain features are extracted from the signal itself. Some of them are based on simple statistic indicators, such as the mean, standard deviation, skewness, kurtosis, peak, and root mean square, among others. Time series models can also be used, such as AR, MA, or the combination of both in an ARMA representation. Dynamic features are also employed depending on the characteristics of the signal, for example: overshoot, settling time, or rise time.

Frequency domain features are obtained from the frequency representation of the signal, most commonly after its Fourier transformation. The same statistic indicators as before can be obtained from the signal in this domain, as well as signal energy or some specific frequency peaks’ amplitudes. Their main limitation is that the signal considered needs to be stationary. To overcome this limitation, time–frequency features can be obtained, which can be obtained through a short-time Fourier transformation, where the analysis of the frequency domain is performed at several time windows [110] or other types of transformations, such us the Wigner–Ville transformation [111] or wavelets [71].

Two-dimensional data such as images require specific feature extraction. These features can be focused on the colour, the texture, the intensity, or the shape, among others [112]. They can also be related to text recognition, as they are useful for traceability purposes in manufacturing.

A summary of the features can be found in Table 2.

### 3.4. Dimensionality Reduction and Feature Selection

The feature selection step is crucial as the dimensionality of the data acquired in manufacturing plants and the amount of features extracted from them are usually high. Ideally, the users of the features would prefer a reduced number of significant features for conducting predictions, which are at the same time interpretable. However, the trade-off between the predictive performance and interpretability is infrequent [108]. Thus, the common solution is to apply some feature selection techniques to reach a compromise. This will also help avoid overfitting [113], which is a general problem found in machine learning applications, related to a model that performs very well in the training set, but is not easily generalised to a different set.

In that sense, techniques can be categorised as supervised or unsupervised, depending on the usage of the labels of the target variables [69]. In supervised techniques, the performance of adding or removing a variable is assessed for predicting the target variable, whereas unsupervised techniques use statistical tests to determine if the new features are similar (redundant) or bring new important information. If no labels are available, the second group should be used, whereas if labels are available, its use would be beneficial not to remove features that may not contain useful information in a particular condition, but may be critical for fault detection.

A very common example inside the unsupervised techniques is Principal Component Analysis (PCA) [114], which was used in [115,116]. PCA can be considered both in the feature extraction and in the feature selection groups, as it involves feature transformation (extraction), but when used, it usually intends to also reduce the dimensionality of the features involved, keeping only the transformed features explaining the most variance. Some nonlinear variants of PCA also exist and have also been used, the most common one being kernel PCA [117]; another one can be found in [118].

Another technique is the minimal Redundancy–Maximal Relevancy (mRMR), developed by [119]. The goal of this method is to find those indicators that minimise the redundancy of the data, as the removal of one feature from highly mutually dependent sets will not lead to a change in the information given by them; at the same time, the method must maximise the relevance to the target classes. There is an unsupervised version of this algorithm (UmRMR) that has been used for predictive maintenance in rotating machinery [120] and in structural health monitoring [121].

## 4. Data Fusion Solutions

### 4.1. Fusion Solutions for Various IoT Environments

The smart manufacturing sector requires underlying enabling technologies in various environments (Table 3). Furthermore, fusion techniques being deployed in these environments comprise the higher computing layer. Next, these environments are described in detail and can be either distributed, heterogeneous, nonlinear, or object-tracking, distributed in terms of the lack of central control, heterogeneous in terms of variety in the device types that should communicate under the same common protocols, non-linear in terms of the existence of time-varying sensing processes, and finally, object-tracking in terms of correct object localisation and identification.

Data sensing technologies are associated with challenges related to distributed environments [122] as in WSNs, which comprise an integral part of the IoT. Since there is heterogeneity in the nature of IoT environments, nonlinear and tracking issues emerge such as Multi-Target Tracking (MTT), cost effectiveness, errors requiring mitigation, and other asynchronous and track-to-track (T2T) problems. Data fusion methods confront all these issues and challenges.

Fusion in distributed environments is a concept that has been confronted in the last two decades. Concerning IoT environments, practical deployment is still an on-going process. Sensors and microprocessors provide source data for the sub-branches of parallel algorithms in such distributed environments. These are the main components used for tracking environmental changes in remote areas. At the same time, microprocessors provide fusion capabilities to them.

Next, classification of fusion algorithms in WSN environments (subset of the IoT) follows. The Kalman Filter (KF) [123] is an estimation algorithm (prediction corrector) and is utilised due to its scalability. It is useful during state propagation and the update of primitive input data. The KF is a Bayesian fusion algorithm and is practically utilised in weather and environmental monitoring, surveillance areas, as well as in intelligent triage systems for tracking sensitive patient data. Since WSNs are dynamic in nature, during operation, power failure issues may arise, connectivity may be unstable due to environmental factors, and sensors can undergo geographical mobility, altering the way of the network’s logical topology. On the other hand, most fusion algorithms exhibit static behaviour.

For WSNs to be cost efficient, they must also be energy efficient. Having a large number of sensors operating together, power instability can cause functional instability at the algorithmic level. A Cuckoo-Based Particle Approach (CBPA) [124] can provide optimisation capabilities in a distributed WSN. This approach is utilised for node deployment in a static cluster. Furthermore, data are aggregated and forwarded to the base station when the cluster heads are selected. This is a generalised swarm algorithm. Next, the Generalised Particle Model Algorithm (GPMA) [125] assists in confronting the energy consumption problem. Specifically, it reduces the complexity by optimising the process of cluster formation with the goal of allocating optimal paths (concerning the reduction of energy consumption) to the base station.

A typical method for reducing energy consumption in many different network architectures (including WSNs) is putting a number of non-passive components into sleep mode when their provision of computing resources is not required. Specifically, sleep mode [126] is used for multimedia sensors in order to save energy. These are only activated by some scalar sensors, which always remain active. The system recognises objects using image analysing techniques, and the results are fused for increasing the recognition performance of sensors.

A cluster formation scheme for energy cost reduction of the data fusion process has been proposed [127]. Due to the limited resources of multisensor schemes, scheduling is a key point for energy efficiency, so a novel hybrid technique has been presented for performing clustering and selection simultaneously. The next step after the selection is the partitioning and processing of data. Blind broadcast messages, along with signal overhead, are reduced during the formation. Next, routing is applied according to the layered architecture with the goal of path elongation minimisation.

Multi-hop routing is an energy-efficient scheme since it utilises traffic grooming techniques for efficient allocation of resources such as bandwidth. Non-passive components are reduced this way along the routing paths. Another technique is to use progressive data fusion, which hops through sensors. This results in low energy consumption. Finally, network planning (predetermination of energy using integer linear programming formulae) also results in reduction. Channel state information and prior knowledge of the routing tree is required during planning.

The basic trait of most fusion IoT systems is the presence of heterogeneity. Various types of devices or sensors interoperate under common communication protocols to assist in fulfilling a specific functionality. An important issue these systems face is the presence of different feature spaces of datasets. Several different spaces comprise the datasets. A challenging task becomes the analysis of the correlations of different data, even when semantic dependencies exist among them.

A framework was proposed [128], aiming at unifying multiple entity views with the purpose of learning embeddings for entity alignments. The views of entity names and their relations and attributes assist in embedding entities, under different combination strategies. The alignment of two different Knowledge Graphs (KGs) is performed by cross-KG inference methods. KG construction and fusion are facilitated by entity matching or resolution. Therefore, entities in different KGs with the same identity are found.

Distributed filtering for the state space models in networking has also been studied [129]. A Bayesian model has been formulated, which handles outliers and heavy-tailed noises, with the purpose of improving the robustness of the filter. A centralised algorithm has been proposed that is based on variational Bayesian methods, providing robust filtering. Next, it has been extended to include the Alternating Direction Method of Multipliers (ADMM). The purpose is to estimate the states and noise covariances.

Schemes have been proposed for managing heterogeneous bio-medical data [130]. A real-time health monitoring system collecting data from the body using sensors has been proposed. A predictive model that is trained on clinical data is applied for detecting malfunctions and generating warnings accordingly. Heterogeneous sensor systems benefit from hybrid fuzzy logic-based algorithms with the Kalman filter. Underground risk has been assessed by a proposed hierarchical fuzzy logic model [131]. For that purpose, two new rule designing and determination methods were presented and evaluated, i.e., the Average Rules Based (ARB) and Max Rules Based (MRB).

Canonical Correlation Analysis (CCA) assists in the analysis of two heterogeneous sets of variables for extracting features that are correlated. In this method [132], there is performance improvement and reduction in computational cost, by optimally predicting the dimension of multimodal information. Next, it was verified that the different types of canonical correlation analyses are special cases of the proposed method, leading to a unified framework. The performance of CCA concerning the fusion output is affected negatively by noisy datasets. When there is dependency between two sets of variables, the Partial Least-Squares (PLS) regression method can be applied. Furthermore, it is applied when the first set contains variables explaining the ones in the other set. In [133], PLS was used to overcome multicollinearity by performing feature extraction. Since PLS is a supervised learning task, it finds orthogonal directions using response values.

In the work of [134], the multimodal fusion of visual and textual similarities was explored from the perspective of an unsupervised framework. These similarities are based on visual features and concepts, as well as textual metadata with the purpose of integrating nonlinear graph-based fusion and PLS regression. A multimodal contextual similarity matrix was constructed, along with the nonlinear combination of relevance scores using query-based similarity vectors.

Multisensor data fusion is affected by nonlinear time-varying sensing processes, producing less-accurate predictions. Mitigating such conditions, optimised algorithms are required for each separate fusion channel. The Extended Kalman Filter (EKF) is extensively used in nonlinear environments. Accuracy improves by using the unscented Kalman filter [122], which is enabled by utilising approximation statistics. The main disadvantages the EKF relate to the linearisation, which is responsible for producing filter instability, and it also requires the existence of the Jacobean matrix. Finally, due to the difficulty of deriving Jacobean matrices, linearisation is a process that is not easily implementable.

The main traits that contribute to the popularity of KF methods relate to simplicity and easy implementation. Outliers inhibit the performance of these filters when sensor-dense IoT networks are operating, resulting in KF breakdowns. Accuracy is not always feasible when AI methods are utilised due to the heuristic nature of the functionality they provide, especially if a large number of sensors is present.

An important research issue is confronting the problem from a Bayesian probabilistic perspective. Using the measurement data, statistically tracking the mean and covariance leads to the estimation of unknown state variables [135]. In such nonlinear networking environments, fusion methods based on fuzzy logic are useful due to the ability to fuse raw data coming from sensors according to predefined rules. These systems can function with high bandwidth and accuracy with sensitive applications. There is also complexity for the update functionality in nonlinear systems [136].

The estimation of a process with an uncertain state with the measurement of noise covariances in nonlinear systems was the research goal of the authors in [142]. For that purpose, an adaptive fusion algorithm with Bayesian cubature was proposed, which jointly estimates system states and covariances with cubature sampling. The goal was to derive the variational Bayesian cubature KF.

The assembly line was assisted by computer vision systems based on object recognition techniques in [137]. These techniques ease the process of identifying complex components and dictate to the operating entity the right task to initiate next.

Data fusion techniques are widely used for the purpose of object tracking. Typical related applications are in robotics and in military equipment and transportation. Specifically, correct target localisation in the military field is facilitated by object tracking techniques. Furthermore, this process involves the detection of threats, the detection of moving objects in restricted areas, as well as timely decision-making.

Object tracking challenges consist of MTT, Track-to-Track issues (T2T), error mitigation, and cost effectiveness. MTT methods rely on data associations that are scene-adaptive. Features of specific targets are accurately determined by exploiting adaptive traits. Data associations take place in hierarchical spaces with different layers.

An example of multi-targeting tracking for an end-to-end transportation network was exhibited in [138]. In this work, an optimal set of trajectories was modelled by generating a graph when exploring deep features. Statistical similarity metrics were utilised for finding the transition cost between nodes. MILP approaches aid in solving the optimisation problem. Another work [139] by the previous authors was related to a hierarchical feature model in the same research field. The proposed algorithm relies on deep features for modelling the appearance of the targets. Next, the tracking problem is confronted by utilizing the unsupervised dimensionality reduction of sparse representation of the feature vectors. Finally, the target is associated by discrete combinatorial optimisation using a Bayesian filter.

The Gaussian mixture probability hypothesis density filter was used for designing an online multi-object tracking method in [140]. In this work, two modules formed the basis for the hierarchical data association, i.e., detection-to-track and track-to-track. These can recover lost tracks and switched IDs. Occlusion problems were also addressed.

Energy efficiency for target tracking in sensor networks is an evolving research field. The authors of [141] presented an algorithm for prediction-based opportunistic sensing in this network type. Sensor nodes self-adapt to target trajectories. They used prediction methods to detect the arrival of targets in close range, so as to put the devices in high consumption mode. When the target was undetected, low-power mode was enabled.

The generic enabling technologies were reviewed for performing fusion in IoT environments. Distributed environments are required when centralised control is not feasible due to the lack of computing and communicating resources. Heterogeneity cannot be discarded since the CapEx and OpEx should remain low. Furthermore, nonlinear and object tracking environments should be confronted as well. In the next section, data fusion for manufacturing environments is described in detail. Some of the techniques used in the general IoT domain can be also found in manufacturing applications.

### 4.2. Data Fusion Solutions in the Manufacturing Sector

In this section, a review of the fusion methods used to combine the heterogeneous sensors employed in industrial prognosis is presented. As mentioned earlier, in machine learning applications, fusion refers to ensemble learning; it is used to optimise the results of a prediction algorithm, and it is categorised according to the stage of the pipeline that it is implemented. Data fusion combines data at an early level; feature fusion is used to describe the combination of extracted features; late fusion describes the combination of the results of different models.

A thorough overview of data fusion systems in the industrial sector can be found in [4]. The authors categorised the industrial scenarios according to the target of the analysis, which affects the features extracted and the type of fusion adopted. The workflow of “data-driven industrial prognosis”, also referred to as the Cross-Industry Standard Process for Data Mining (CRISP), mentioned data fusion and preparation as a middle step in the analysis’ pipeline, before proceeding with the modelling and evaluation. Data fusion exploits additional sources of information other than those that already existing in an industry unit, such as environmental factors, which can provide important added value.

In [71], information fusion theory was adopted to combine features in order to deal with the nonlinear problem of machine condition recognition. The sensors used were a dynamometer for cutting force signals and an accelerometer for vibration signals. The abnormal conditions that needed to be recognised were tool wear and workpiece deformation. Before applying feature fusion, signal processing took place. Fast Fourier transformation and then discrete wavelet transformation were used to process the signals and extract features. Approximation and detail components were extracted from the signals and used as the input for the analysis. Cutting parameters and signal characteristics were fused in one characteristic vector with concatenation, and then, Support Vector Machine (SVM) models were applied to recognise the different machine conditions. In [143], the authors performed tool wear prediction and fused multi-domain features with the use of deep neural networks. Cutting force and vibration sensors were used to monitor the tool wear condition. Time, frequency, and time–frequency domain features were extracted from these sensors. The ground truth about the condition of the tools was obtained through a microscope in offline mode. The authors normalised the values of the extracted features and the target values, before applying the Deep Convolutional Neural Network (DCNN) model.

Surface quality control was performed in [72]. Four methods for signal processing were used, and a multisensor data fusion framework was presented. The cutting forces, acoustic emission, and vibration sensors were used to monitor the quality of the surface. Time Direct Analysis (TDA), Power Spectral Density (PSD), Singular Spectrum Analysis (SSA), and Wavelet Packet Transform (WPT) were the four feature extraction methods applied to the sensor data to extract statistical and non-statistical parameters. The multisensor fusion framework refers to the combination of parameters of all available sensors and of cutting forces and vibration sensors only. The framework was examined separately for each feature extraction method and for TDA and PSD together.

A more complex approach for data fusion in two stages was proposed in [144]. The authors dealt with the problem of condition monitoring. The fusion approach was based on Bayesian inference. In the first stage of the fusion framework, the data were combined at a local level, which in the particular application translates into individual health components. The obtained results of this level were used as the inputs in the second stage, which was the global one, and the condition of the whole system was assessed. The authors extracted a variety of time and frequency domain features from the time waveform of the signals. A two-stage Bayesian method combined with PCA was used as a sensor fusion scheme in [115]. The authors combined vibration, electric, and acoustic signals for mechanical and electrical fault diagnosis in induction motors. PCA was used to remove correlated features extracted from the three types of sensors. At the first stage of the Bayesian approach, the principal components of the features were combined with a Gaussian Naive Bayes (GNB) classifier, and the fusion of the local stages’ results followed in the second (global) stage of the approach.

In [79], the authors performed CNC machining monitoring with the use of three sensors and also proposed a data fusion framework that improved the monitoring. Microphones were placed at three different locations in order to receive measurements for cutting parameters. The fusion framework comprised the typical procedure of signal extraction, filtering with the use of band-pass filters, and normalisation. Afterwards, using the autocorrelation coefficient, preference weights were calculated, which added a bigger weight to the preferred sensor. Finally, a signal estimate was formed by the sensor with the highest weight and the maximum likelihood estimate. This proposed fusion scheme is suitable for periodic transient signals.

In [44], Deep Neural Networks (DNNs) were used for sensor data fusion and analysis. The data under analysis comprised heterogeneous sensors attached to a replica industrial plant. The goal of the analysis was to assess the working conditions of the industrial plan with a fault prediction algorithm. The sensors involved were a vibration, proximity sensor, temperature, audio (measuring the noise produced by the machine replica), and current sensor. Each of these sensors measures a different condition of the plant. The authors faced the problem of monitoring all of these conditions as a multiclass classification problem, where each of the classes refers to the state of a condition. The fusion of the four sensors, excluding temperature, was performed with a DNN. The sensors had different sampling frequencies. In the preprocessing stage, the outliers were removed. On the audio signals, fast Fourier transformation was applied to extract frequency features. From the current and vibration sensors, the maximum values were extracted using time windows of different lengths for each sensor. No features were extracted from the proximity sensor. All these features were combined using a concatenation layer in the DNN. Deep fusion and feature level fusion were combined in [145] to perform fault severity diagnosis under various operating conditions. Multiple sensors, such as torque sensors and accelerometers, were installed on an experimental conditioning platform, to test the proposed framework. The signals provided by the sensors were segmented, and the features were extracted by each segment separately. The features of different segments were combined and formed a single feature vector. Afterwards, with the use of the DNN, the features were deeply fused in the layers of the network. After the deep fusion stage, the training and testing of classifiers followed.

A DCNN was found in [146]. The authors proposed an adaptive fusion method based on DCNNs that addressed the issues of multisensor feature extraction and the selection of a suitable fusion method in terms of the stage in which it is performed. The capability of DNNs to fuse data into different layers and stages was the reason the authors adopted them in their approach. The adapted network fuses the input data at lower levels and then extracts basic features, which are then, in the middle layer, fused into high-level features and decisions, and finally, at the higher levels, the features and decisions are combined again to obtain the final prediction.

A quite recent paper [74] dealing with the problem of milling chatter detection proposed a multisensor data fusion scheme to combine accelerometer and microphone inputs. After creating chatter features with Wavelet Package Decomposition (WPD), the authors performed parameter optimisation, then identified the WPD coefficients that were correlated to the resonant frequencies, and extracted time–frequency features. The fused features of both sensors were selected with the application of the Recursive Feature Elimination (RFE) method.

In [80], the authors applied a random-forest-based fusion to combine vibration and acoustic signals for fault diagnosis. An accelerometer was used to provide the vibration signals and an acoustic emission sensor for the acoustic signals. The sensors were operating at the same time to monitor the condition of a gearbox. The authors applied wavelet packet transform to extract the statistical parameters of the two sensors, which were later fed into two deep Boltzmann machines, respectively, to extract the deep features. Then, the features were fused using random forests.

In an older paper [147], fault diagnosis was again performed with acoustic and vibration signals, which were combined using Dempster–Shafer theory. This fusion was performed at the decision level. The signals from the two sensors were preprocessed using wavelet analysis, and the extracted data were used as the input in artificial neural networks for classification. The results of the networks were then combined using Dempster–Shafer rules, and the fusion improved the individual performance of the sensors per 10%. Late fusion was also used in [148] to improve the diagnosis of bearing defects in induction motors. The authors first combined the features extracted from the vibration sensors, after removing the redundant ones. Afterwards, they tried four different classifiers, which were later combined using ensemble methods, such as voting and stacking.

Feature fusion with the use of kernel techniques was presented in [149]. The authors referred to the term “fusion” along with feature selection as a technique for dimension reduction. The authors combined kernel theory with factor analysis and proposed a Probabilistic Kernel Factor Analysis framework (PKFA), employed for feature selection and fusion in tool condition monitoring. After the acquisition of data from vibration and force sensors involved in the machine processes, feature extraction was applied to extract statistical and frequency features, which measure the health condition of tools. The proposed PKFA was then applied to select and fuse features. For the prediction of tool wear condition, support vector regression was used.

The above mentioned methods are summarized in Table 4, categorized according to the stage of the analysis they are performed.

## 5. Discussion

In this paper, an overview of recent trends in sensor data analysis and fusion applications in the manufacturing sector was provided. The reviewed literature was categorised according to the stages of the analysis. First, data storage solutions were presented, including acquisition methods and communication protocols. Afterwards, preprocessing methods employed in the manufacturing sector were presented. The preprocessing steps of sensor data analysis demand much effort. Considering that most of the acquired data in industrial prognosis are sensor signals, filtering and feature extraction should be applied to eliminate noise.

The current literature review presented first of all the data storage and extraction solutions used in the sector. The vast variety of solutions that were presented are used in order to create a pipeline capable of handling the data from acquiring to storing. Most of them involve a wireless method for gathering data and a database solution capable of storing the data efficiently. Due to the nature and volume of the data, distributed databases are preferred.

Fusion methods are increasingly involved in sensor data analysis in manufacturing applications. From the literature reviewed, it can be stated that data or feature level fusion are more frequently applied in industrial prognosis. It can be also noted that the term data fusion was found to be used broadly to describe the tasks of feature extraction and feature selection, where the features of different sensors were combined using mainly concatenation. Fewer were the applications of late fusion methods, although there is a wider variety of them compared to early fusion ones. DNNs are a popular fusion method in the manufacturing sector, combining different sensors by addressing them in different layers and also offering the advantage of skipping the step of feature extraction.

Regarding signal processing and feature extraction, the computational efforts required by the algorithms imply some handicaps in the implementation of these strategies. In contrast, the use of extremely straightforward techniques can lead to low-quality information for the purposes of the manufacturing industry. Therefore, a compromise is required between the duration of the running time of the processing and feature extraction algorithms and the quality of the data being used for posterior analysis.

There is still room for experimenting with different fusion methods in the field of manufacturing, considering the more narrow variety of fusion methods adopted, compared to other fields. According to this review, as well as other reviews regarding sensor data analysis in the manufacturing sector, the combination of heterogeneous sensor data is mostly achieved through concatenation or DNNs. Real-time applications of prognosis, such as chatter or tool wear detection, definitely affect the type of fusion adopted, while off-line implementations allow for more experimentation. There are of course numerous late fusion methods that are not time demanding and can be employed in a real-time application. The implementation of more sophisticated fusion methods is one possible research direction for further investigation. Researchers should take into account the issues of large data volumes extracted from sensors and the time complexity of feature extraction and of algorithm implementation.

In order to apply efficient fusion solutions in the manufacturing sector, the underlying network technologies should be considered as well. Production environments rely on IoT connectivity, among other technologies, for energy efficiency and performance. Spectrum allocation is a process where machine learning tools emerge for improving adaptivity to the runtime conditions, when embedded in the computational logic and communication protocols. These tools are applied upon the enabling technologies of IoT networks. An overview of the aforementioned enabling technologies was performed, as well as the correlation with distributed, heterogeneous, nonlinear, or object-tracking environments.

It should be highlighted that the conception of all the technologies described in the paper followed its own path and that their strengths were combined in order to generate analysis workflows as the one described in the Introduction. Having said that, the adoption of these technologies by the industry does not follow a homogeneous trend. The differences between sectors (in terms of competitiveness, required quality standards, etc.) generate different needs when it comes to digital transformation and, specifically, multisensor data fusion. Moreover, some companies opt for the application of a subset of the aforementioned workflow, by the use of portable equipment with sensing, signal processing, and feature extraction capabilities for non-skilled workers that can be enough to monitor some of the critical points of the manufacturing plants. Apart from that, the differences with respect to investment capabilities and skills of the personnel of the companies in the specific technologies generate also a heterogeneous adoption of these technologies.

Thus, the purpose of this literature review was to present the recent trends in data fusion solutions for industrial prognosis, specifically for the manufacturing sector. This paper aims to serve as a practical guide for those interested in learning about methodologies and trends in analysing manufacturing sensor data, as well as the more advanced technique of fusion.

## Figures and Tables

**Table 2:** Summarised features according to the domain.

Domain	Features	Reference
Time domain	Mean, maximum, minimum, amplitude, variance, standard deviation, skewness, kurtosis, root mean square, peak-to-peak, autoregressive coefficients, overshoot, settling time, rise time	[73,98,109]
Frequency domain	Spectral statistic moments, highest peak amplitude, sum of peak amplitudes	[73,98,105]
Time-frequency domain	Wavelet energy, Wigner–Ville	[71,110,111]
Two-dimensional domain	RGB, LUV, HSV, HMMD, homogeneity, entropy, contrast, correlation, body shape, length, width, height	[112]

**Table 3:** Fusion environments.

Environment	Subfield	Reference
Distributed	Data sensing	[122]
	Kalman filtering	[123]
	Energy/cost efficiency	[124,125,126,127]
Heterogeneous	Data correlation	[128]
	Distributed filtering	[129]
	Heterogeneous data	[130]
	Fussy logic/Kalman filter	[131]
	Canonical correlation analysis	[132,133]
	Multimodal fusion	[134]
Non-linear	Multisensor data fusion	[122]
	Sensor-dense IoT networks	[135]
	Fusion based on fuzzy logic	[136]
Object-tracking	Assembly line	[137]
	Transportation network	[138,139]
	Online multi-object tracking	[140]
	Energy efficiency for target tracking	[141]

**Table 4:** Summary of the fusion methods.

Fusion Level	Fusion Method	Reference
Feature	Information theory	[71]
Feature/late	Bayesian-based fusion	[115,144]
Feature	D(C)NNs	[44,143,145,146]
Feature	Feature elimination/concatenation	[72,74,79,149]
Late	Dempster–Schafer theory	[147,148]
Feature	Random forest based	[80]

## Data Availability

Not applicable.

## Abbreviations

The following abbreviations are used in this manuscript:

ICTs	Information Communication Technologies
IoT	Internet of Things
WSNs	Wireless Sensor Networks
RFID	Radio Frequency Identification
DAQ	Data Acquisition Device
RF	Radio Frequency
OPC-UA	Open Platform Communications Unified Architecture
SQL	Structured Query Language database
CPS	Cyber–Physical System
MES	Smart Manufacturing Execution System
IPv6	Internet Protocol Version 6
MQTT	MQ Telemetry Transport
SOAP	Simple Object Access Protocol
REST	Representational State Transfer
SCADA	Supervisory Control And Data Acquisition
ODBC	Open Database Connectivity
DDE	Dynamic Data Exchange
RS232	Recommended Standard 232
HART	Highway Addressable Remote Transducer
API	Application Programming Interface
HTTP	Hypertext Transfer Protocol
LE	Low Energy
NFC	Near-Field Communications
LPWAN	Low-Power Wide-Area Network
LoRa	Low Range
NB-IoT	Narrowband IoT
CNC	Computerised Numerical Control
TCP/IP	Transmission Control Protocol/Internet Protocol
UHF	Ultra-High Frequency
IIoT	Industrial Internet of Things
DDBS	Distributed Database System
UHF	Ultra-High Frequency
IIoT	Industrial Internet of Things
DDBS	Distributed Database System
HDFS	Hadoop Distributed File System
COSMOS	C Open Source Managed Operating System
GFS	Google File System
PDU	Protocol Data Unit
DBMS	Database Management Systems
OLTP	Online Transaction Processing
RDBMS	Relational Database Management Systems
DO-SS	Document-Oriented Storage System
OO-SS	Object-Oriented Storage System
SDHC	Software-Defined Hybrid Cloud
ISO	International Organization for Standardization
VDI	Verein Deutscher Ingenieure
AE	Acoustic Emission
MCSA	Motor Current Signature Analysis
PCA	Principal Component Analysis
mRMR	minimal Redundancy–Maximum Relevance
CRISP	Cross-Industry Standard Process for Data Mining
SVM	Support Vector Machine
DCNN	Deep Convolutional Neural Network
GNB	Gaussian Naive Bayes
DNN	Deep Neural Networks
WPD	Wavelet Package Decomposition
RFE	Recursive Feature Elimination
PKFA	Probabilistic Kernel Factor Analysis
